# Selective Feeding—An Under-Recognised Contributor to Picky Eating

**DOI:** 10.3390/nu16213608

**Published:** 2024-10-24

**Authors:** Terri X. B. Chiong, Michelle L. N. Tan, Tammy S. H. Lim, Seng Hock Quak, Marion M. Aw

**Affiliations:** 1 Department of Paediatrics, Khoo Teck Puat-National University Children’s Medical Institute, National University Hospital, Singapore 119228, Singapore; 2 Yong Loo Lin School of Medicine, National University of Singapore, Singapore 117597, Singapore

**Keywords:** picky eating, feeding difficulties, selectivity, caregiver feeding style, growth and nutrition

## Abstract

*Background*: Amongst children presenting to an interdisciplinary clinic with complaints of picky eating, we aim to identify the proportion who have underlying selective feeding and to describe its implications on growth and nutrition, as well as parental coping responses. *Methods*: We conducted a retrospective chart review of first-visit consults from January 2020 to July 2022. Caregiver and child mealtime behaviours were assessed using the standardised Caregiver’s Feeding Styles Questionnaire (CFSQ) and by direct observation. Caloric intake and oromotor skills were assessed by dietitians and speech therapists, respectively. Medical concerns were addressed by the doctor. *Results*: Out of 152 children referred for concerns of “picky eating”, 128 (84.2%) were diagnosed as having selective eating, while the rest were diagnosed with delayed oromotor skills, poor appetite, oral aversion and 4 were deemed to have normal feeding behaviour for their age. Of the 128 selective eaters, 67 (52%) children had comorbidities such as autism spectrum disorder (ASD) (*n* = 59), attention-deficit/hyperactivity (ADHD) (*n* = 2) and underlying medical conditions (*n* = 6). The remaining 61 children were “otherwise well”. Of the “otherwise well” children, 47.5% had inadequate caloric intake and 31% had failure to thrive. The commonest feeding style among caregivers of “otherwise well” picky eaters was authoritarian (36%). The majority (80%) of these caregivers also experienced helplessness. *Conclusions*: We conclude that picky eating in young children is a symptom with several possible underlying aetiologies. It is associated with nutritional consequences for the child and significant stress on caregivers. Being able to recognise those who need referral for specialist intervention and multidisciplinary management (such as selective feeding and delayed oromotor skills) would be important.

## 1. Introduction

Picky eating is a common concern among caregivers of young children. The reported prevalence can vary from below 10% to more than 50%. In a local questionnaire survey, as many as 49.6% of Singaporean caregivers perceived that their children aged 1 to 10 years are “picky eaters” [1]. This variation in prevalence could be due to the inconsistent definition of “picky eating”, different age groups in different studies, or even cultural differences [2]. Picky eating may refer to the consumption of a limited variety of foods, having strong food preferences, being unwilling to try new foods, or even consuming a limited amount of food. The various definitions may also vary in severity from being a mild and transient problem to one that results in nutritional deficiencies. Different studies have also used different ways of assessing it, for example, caregiver report, validated questionnaires (e.g., Child Feeding Questionnaire, Child Eating Behavior Questionnaire, etc.), clinical assessment by professionals, or even the use of a single question (for example, “does your child have definite likes and dislikes as far as food is concerned?”). Whilst it is not unexpected that young children may have developmental neophobia, children with underlying textural hypersensitivity, poor appetite or delayed feeding skills could also present as “picky eating” to the primary care physician. These latter conditions would require prompt specialist attention rather than a watchful waiting approach [3]. Recognizing the underlying issues driving the picky eating is important to tailor appropriate therapy for each child.

Left untreated, feeding difficulties over time could negatively impact a child’s growth and nutrition. Children whose parents expressed concern about their feeding had a three times higher risk of poor growth [4]. This poorer growth (weight, height) may persist beyond childhood, into adolescence [5,6]. More severe feeding difficulties and food selectivity, such as those seen in children with autism, have been reported to result in nutritional deficiencies in iron, calcium and, vitamin D [7,8]. Beyond the child, feeding difficulties often result in significant caregiver stress [1]. It may negatively impact the parent–child relationship, family relationships as a whole and even limit the family’s social behaviours and social participation [9]. As feeding is an interactive process between the caregiver and the child, the caregiver’s response to the stress of feeding, as well as the child’s feeding behaviours, could have a reciprocal effect on each other [10,11,12].

Our current study aims to identify predisposing factors or underlying aetiologies in children referred to an interdisciplinary feeding clinic for concerns of “picky eating”. We also describe the dietary habits of the picky eaters and the consequences on their growth and nutrition, as well as the emotional impact picky eating has on their caregivers. We believe that this study will increase awareness amongst general paediatricians and primary care physicians to the issue that picky eating may not be something that a child “would grow out of”, and that some would require referral and management by an interdisciplinary feeding team.

## 2. Methods

### 2.1. Study Settings and Participants

This is a retrospective chart review of first-visit consultations from January 2020 to July 2022 to an interdisciplinary feeding clinic located in one of two government hospital providing paediatric services in the country. This interdisciplinary team comprises paediatricians, paediatric gastroenterologists, speech therapists, dietitians and psychologists. Inclusion criteria were (i) age 0–18 years; (ii) concerns of “picky eating” by the referring physician or by the child’s parents. Children who were tube-fed, had incomplete dietary data, or had incomplete oromotor skill assessment were excluded.

Following the consultation, each child was assessed according to the Pediatric Feeding Disorder Consensus Definition and Conceptual Framework by Goday et al. [2]. Paediatric feeding disorder is defined as impaired oral intake that is not age-appropriate, and is associated with medical, nutritional, feeding skill, and/or psychosocial dysfunction, lasting for at least 2 weeks. In addition, we also evaluated if the patients had any underlying cause of their feeding difficulties, including delayed oromotor skills, poor appetite, oral aversion, selective feeding or if the parent had misperceived the child to have feeding difficulties (i.e., the child has age-appropriate feeding skills and behaviours). Delayed oromotor skills may present as a child who is unable to consume age-appropriate liquid and food textures. The child may require food or fluid to be modified from its original form that is not age-appropriate. Feeding aversions result when a child repeatedly experiences physical or emotional pain or discomfort during feeding. Over time, the child develops strategies to avoid the aversive feeding situations [13]. Selective feeding is defined as eating a limited variety of foods or unwillingness to try new foods, despite the ability to eat a broader diet [14]. In this cohort, we have defined selective feeding as the intake of 15 or fewer foods [15].

### 2.2. Medical and Nutritional Information

During the clinic visit, the doctor will assess for any medical conditions that may be contributing to the child’s feeding difficulties. Relevant investigations may be ordered if deemed necessary by the doctor. Caloric intake is assessed by the dietitian using a 2- or 3-day caregiver-reported food diary and corroborated by interviewing caregivers during the clinic session. Caregivers may also share pictures of the child’s foods or meals to better understand the child’s diet. Based on the diet history, the dietitian will then calculate the child’s intake of calories, protein, carbohydrate, fat, iron and calcium using Foodworks 10 Professional (an industry standardised nutritional analysis software). Food items from each food group (vegetables, fruits, protein and alternatives, rice and alternatives, dairy and alternatives) as well as beverages that the child is consuming (at the time of the visit) are also clarified with the caregiver during the session.

### 2.3. Oromotor Skills

Oromotor skills are assessed by the speech therapist by direct observation of a meal in clinic, or via video recording of a child’s typical mealtime if direct observation was not available. This was carried out in accordance with age-matched guidelines [16]. Further investigations such as a video-fluoroscopy may be warranted if dysphagia is suspected.

### 2.4. Anthropometric Measurements

Heights and weights were taken for all patients on their first visit by clinic nurses as per standard clinical protocol. Height was measured to the nearest 1 mm using a fixed stadiometer. For children below 2 years of age, supine length was measured using an infantometer. Weight was measured to the nearest 0.1 kg. World Health Organisation (WHO) growth standards were used to determine the child’s weight-for-age and height-for-age percentiles. The presence of failure to thrive is defined in this study as weight less than the 3rd centile for age, or weight crossing downwards over 2 or more major percentile lines. Overweight was defined as weight-for-age more than the 90th centile.

### 2.5. Caregiver’s Feeding Styles Questionnaire (CFSQ) and Caregiver Coping

Caregiver feeding style was assessed using the CFSQ [17,18] and direct observations made in clinic by a psychologist and speech therapist. The CFSQ is a validated questionnaire describing the behaviours that caregivers demonstrate during the child’s mealtimes, based upon their degree of “responsiveness” and “demandingness” towards the child [19]. Demandingness represents how much a caregiver controls what the child eats, while responsiveness represents how caregivers encourage their children to eat. Based on these dimensions, four feeding styles are characterised: (1) Authoritative, where a nurturing feeding environment is provided yet there are set expectations; (2) Authoritarian, characterised by high level of caregiver control and restriction, with less influence from the child’s needs; (3) Indulgent, characterised by acceptance of child’s feeding behaviour with few expectations; (4) Uninvolved, where there is little caregiver involvement with the child’s feeding.

Caregiver coping was assessed by observation of the caregivers’ interaction with the child during a feeding session, by a paediatric psychologist. The following terms were used by the psychologist to describe the interaction: helpless, frustrated, anxious, emerging, confident. The term “emerging” meant that the parents/caregivers were developing competence and confidence in feeding.

### 2.6. Statistical Analysis

The association between potential aetiological factors and the dichotomous groups of neurotypical children versus those with ASD was explored using Chi-Square tests and two samples *t*-tests for categorical and parametric data, respectively. A 5% level of significance (alpha) was used, and 95% confidence intervals were presented. A multi-variate logistic regression was further performed to explore the magnitude of association between “selective feeding behaviour” and its attendant co-morbidities to control for potential confounding covariates. Data were analysed by using SPSS Statistics for Windows, Version 23.0 (IBM SPSS Statistics for Windows, Version 23.0. Armonk, NY, USA: IBM Corp.).

This study was conducted according to the guidelines of the Declaration of Helsinki, and approval for the study was obtained from the Singapore National Health Group Domain Specific Review Board (DSRB) (2019/00828).

## 3. Results

A total of two hundred and fifty-nine new patients were referred in this time period, of which one hundred and fifty-two (58.7%) were for picky eating. The median (range) age at presentation was 28 (3–163) months.

### 3.1. Underlying Aetiologies of Picky Eating

Following comprehensive assessment (as described above), the underlying aetiologies for the 152 children presenting with “picky eating” are shown in Table 1. In this group, the majority (84.2%) exhibited selective feeding behaviours.

### 3.2. Comorbidities Amongst Children with Selective Feeding

**Figure 1:**
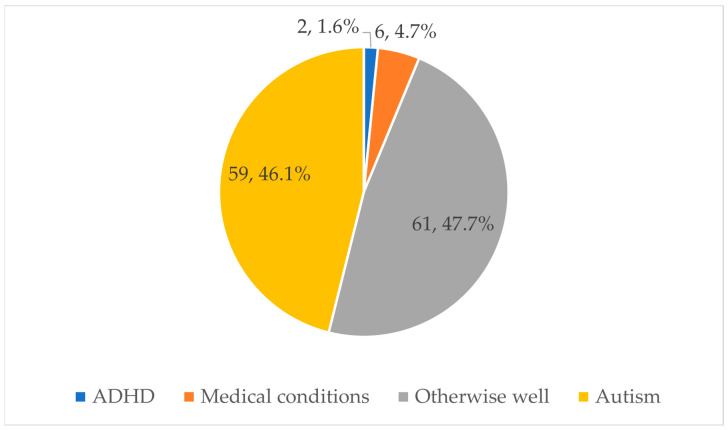
Co-morbidities amongst children with selective feeding.

Sixty-seven (52%) children had comorbidities including autism spectrum disorder (ASD) (*n* = 59), attention-deficit/hyperactivity (ADHD) (*n* = 2), and other medical conditions (*n* = 6) (Figure 1). The medical conditions included epilepsy (2), eosinophilic esophagitis (1), previous gastroesophageal reflux disease (1), poor exposure to food in infancy secondary to an early onset oncologic diagnosis (1), and neurodegenerative disease (1). The remaining 61 children were otherwise well, with no significant medical condition.

Having ASD or ADHD was associated with increased odds (5.3, CI: 1.9–14.8) of having selective feeding behaviour.

Children with ASD are well known to have sensory issues. They would naturally be on a physician’s radar for early referral if feeding difficulties or picky eating was flagged by their caregivers. For our study, we focused our attention on the “otherwise well” child with picky eating, and also compared them as a group with the children with ASD.

### 3.3. Dietary Habits of Children with Selective Feeding

Children with ASD accepted significantly fewer foods than the “otherwise well” child (mean of 15.1 foods compared to 11.6 foods, *p* = 0.003) (Table 2), and this was seen across all food groups (Table 3). Children with ASD also accepted fewer food groups than neurotypical children (3.9 versus 4.3 out of 5 food groups, *p* = 0.037), with a larger proportion accepting 10 foods or less (Table 2) (*p* = 0.005, OR 3.0, 95% CI 1.4–6.4).

The most commonly rejected food group was vegetables, and this was similar in both the ASD and neurotypical child. However, children with ASD were 2.7 times more likely to reject fruits compared to neurotypical children [*p* = 0.027, OR = 2.7, 95% CI 1.1–6.7] (Table 3). All of the neurotypical children accepted rice and/or alternatives, with an average of 4.4 foods in that category, whereas 6.8% of those with ASD rejected the food group completely (*p* = 0.055). As a result of their selectivity with food, milk was the main source of nutrition in nine (15%) of the children with ASD (but in only one (1.6%) neurotypical child (*p* = 0.007, OR 10.8, 95% CI 1.3–88.2).

### 3.4. Anthropometric Data of Children with Selective Feeding

The impact of selective feeding on a child’s overall nutritional status and growth can be seen in Table 4 and Table 5. As many as 20–30% of children had failure to thrive. Neurotypical children had significantly lower weight and height Z-scores for their age as compared to their ASD counterparts. This is consistent with the observation that despite having more restrictive diets, children with autism were more likely to consume an adequate number of calories compared to the neurotypical patients (*p* = 0.002, OR 3.5, 95% CI 1.6–7.8), and in fact, a higher proportion of them were overweight (13.6% compared to 4.9%). However, because of their higher selectivity, they were more likely to have an oral intake that was deficient in micronutrients such as iron and calcium, despite an adequate calorie intake (*p* = 0.003, OR = 3.5, 95% CI 1.5–8.2).

Overall, of the children with selective feeding who did not have failure to thrive, more than 40% had inadequate total calorie or micronutrient intake. This signifies the importance of a thorough assessment of their diet even if they seem to be growing adequately.

### 3.5. Caregiver Feeding Styles of Children with Selective Feeding

Sixty-two percent (38 out of 61) of the “otherwise well” children with selective feeding and eighty-three percent (49 out of 59) of the ASD patients were assessed for caregiver feeding styles with the CFSQ and through observation of the feeding session by the speech therapists and psychologists.

The commonest feeding style was authoritarian in both groups (Table 6).

### 3.6. Caregivers’ Coping and Response

Caregiver coping was assessed in 57% of the “otherwise well” children with selective feeding (35 out of 61) and in 80% of children with ASD (47 out of 59).

We found that caregivers had similar emotional responses to their child’s eating behaviours, regardless of whether there was underlying ASD or not, with the majority demonstrating helplessness, frustration and anxiety. Only 28.6% of caregivers of the “otherwise well” children with selective feeding and 15% of those caring for ASD children showed emerging competence. None of the caregivers demonstrated confidence in their feeding interactions with their child (Table 7).

## 4. Discussion

This is the largest reported study of picky eaters assessed and managed in a feeding clinic within Southeast Asia. A significant number of these picky eaters were assessed to demonstrate selectivity (84.2%) in their eating behaviours. Whilst this figure is likely an overestimate of its prevalence in a community setting of picky eaters, it does alert us to its existence and importance as a contributing factor to what primary care physicians may otherwise pass off as something a young child would outgrow, when specific intervention may actually be required.

Among the selective eaters in our study, up to 52% have underlying comorbidities, specifically autism spectrum disorder. However, an almost equally large number did not have any underlying medical co-morbidity. Whilst it is not un-expected that children with ASD have selectivity in feeding, contributed in part by the child’s sensory issues or rigidity in behaviours, these would need to be specifically looked for in children who are “otherwise well”, i.e., sensory or textural difficulties contributing to their selective feeding.

In addition, support for caregivers would be essential [20], in terms of both emotional wellbeing as well as intervention for their child. These children are best managed by an interdisciplinary team consisting of nutritionists to determine if there are any macro- or micronutrient deficiencies in their diet and how to overcome them, developmental psychologists to assist with behavioural modification, and speech therapists to address the children’s sensorial hypersensitivities.

These children with selective feeding, especially when they accept 15 foods or less, or reject whole food groups, are at risk of micronutrient and macronutrient deficiencies. Studies comparing children with picky eating and those without picky eating have found that the picky eating group was more likely to consume insufficient micronutrients (e.g., iron, zinc, vitamin D) [21]. In this study, almost two-thirds of our patients were assessed to have insufficient caloric and/or micronutrient intake, affecting even the ones without failure to thrive. Early therapeutic intervention to encourage expansion of their food repertoires may avoid consequences on their growth and development. Of note, the ASD children were more likely to consume sufficient or excessive calories as compared to the “otherwise well” children with selective feeding, which explains the higher mean body weight in the ASD group. This has been reported in previous literature as ASD patients tended to have selective preferences for high-calorie processed foods, sweets and soft drinks [22].

It is important that the primary physician recognises when picky eaters should be referred for specialist evaluation and intervention. One of the main challenges is that there are a variety of reasons behind why a child is a picky eater. Children with poor appetite or neophobia appropriate for their age may be managed first in primary care with strategies such as caloric fortification of foods, promoting positive associations with feeding and enforcing appropriate mealtime structures.

Some children have a real pathology that needs to be addressed by trained healthcare providers. Selectivity is one such example. In our context, significant food selectivity can be identified if children are eating 15 or fewer different foods or excluding whole food groups. These children would need to be referred. Children with delayed feeding skills who are not eating age-appropriate food textures may also present with picky eating and need to be further evaluated and managed. In addition, children with autism would be best managed by an interdisciplinary feeding team, as feeding difficulties in autistic children tend to have a more severe and protracted trajectory, as compared to typically developing children [23].

Caregivers should also be evaluated for their feeding styles and coping strategies, as these have a major influence over the child’s feeding environment and can maintain or exacerbate difficult feeding behaviours [17]. This may be challenging to do in a busy primary care setting as consultation time is limited. Our study suggests that the commonest caregiver feeding style among the picky eaters is authoritarian (49%), followed by authoritative feeding style (20.7%). This is in contrast to a previous Brazilian study where children with feeding difficulties had an equal proportion of caregivers who had authoritative and authoritarian feeding styles (39.5% each) [18]. The reason for this could be cultural, as Asian caregivers tend to adopt a more authoritarian parenting style. In a previous study from our centre, caregivers of the autistic children were identified to have predominantly an authoritarian (34.8%) or indulgent feeding styles (39.4%). The authoritarian feeding style was associated with increased feeding difficulties [24].

The majority of caregivers also displayed helplessness, which likely undermines their confidence in effecting change in their child’s feeding habits. As part of holistic management of the child’s feeding environment, empowering caregivers with knowledge on appropriate feeding styles, and guiding them with specific steps to take would be essential.

## 5. Limitations

Firstly, this study includes children from an interdisciplinary feeding clinic in a tertiary hospital. These children are likely to have more severe feeding difficulties, hence overestimating the proportion of “otherwise-well” children with picky eating who have underlying sensory (or contributing) issues, compared to a population of otherwise well children in the community. Future studies could be performed to evaluate similar data from general paediatric clinics. Secondly, as the aim of this study is to describe underlying aetiologies contributing to picky eating, only data from the first visit to the clinic were evaluated. The impact of feeding intervention and prognoses of these children are not included and could be the focus of a separate study. Thirdly, the response rate on the CFSQ was 68.3%. This was not 100% for two main reasons: (i) the main caregiver who usually fed the child was not present at that clinic visit, or (ii) the main caregiver did not read or understand English sufficiently to complete the questionnaire. This may affect the generalisability of this study if there are differences in families who speak English versus those who do not. However, we do not believe that cultural or socioeconomic factors would contribute significantly to the presence of underlying drivers of picky eating such as selectivity (which are mainly sensory or textural), but it could certainly contribute towards parenting behaviours in response to that feeding difficulty, or health-seeking behaviours. Whilst the CFSQ has not been formally validated in the Asian context, it has been used in other Asian cohorts, which have similarly reported that the most common feeding style is the authoritarian style [25,26]. Further studies could be undertaken to study the validity of the CFSQ, particularly translated versions of the CFSQ.

## 6. Conclusions

Picky eating in young children is a symptom with several possible underlying aetiologies. It is associated with nutritional consequences for the child and emotional burden on caregivers. Being able to recognise what can be managed in primary care (poor appetite) and what may need referral for specialist intervention (selectivity, delayed feeding skills, autism), as well as the need to support caregivers (emotionally as well as educationally) would be important. Further studies are needed to assess the outcome of these children after interdisciplinary interventions.

## Figures and Tables

**Table 1:** Diagnosis of patients presenting with “picky eating” to the feeding clinic.

Diagnosis	
Selective feeding (*n*, %)	128 (84.2%)
Delayed oromotor skills (*n*, %)	10 (6.6%)
Poor appetite (*n*, %)	9 (5.9%)
Age-appropriate feeding behaviours (*n*, %)	4 (2.6%)
Oral aversion (*n*, %)	1 (0.7%)

**Table 2:** Foods accepted by “otherwise well” children with selective feeding as compared to those with autism spectrum disorder.

	“Otherwise Well” Children (*n* = 61)	Children with Autism Spectrum Disorder (*n* = 59)	*p*-Value
Average number of foods accepted	15.1	11.6	0.003
Number of patients accepting ≤ 10 foods (*n*, %)	15 (24.6%)	29 (49.2%)	0.005
Average number of food groups accepted (out of 5)	4.3	3.9	0.037

**Table 3:** Proportions of “otherwise well” and ASD selective feeders who rejected the following food groups.

Rejected Food Group	“Otherwise Well” Children	Children with Autism Spectrum Disorder	*p*-Value (Chi Square)	Adjusted Odds Ratio (95% CI)
Vegetables	41%	44%	0.733	1.427 (0.625–3.259)
Fruits	14.8%	32.2%	0.024	0.355 (0.135–0.932)
Protein, meat and alternatives	11.5%	18.6%	0.272	0.585 (0.188–1.822)
Rice and alternatives	0%	6.8%	0.039	Not applicable
Dairy and alternatives	3.3%	6.8%	0.379	0.908 (0.137–6.031)

**Table 4:** Anthropometric data of “otherwise well” children with selective feeding as compared to children with autism spectrum disorder.

	“Otherwise Well” Children	Children with Autism Spectrum Disorder	*p*-Value
Number of Patients with Failure to Thrive (*n*, [%])	19 (31.1%)	13 (22.0%)	0.259
- Falling across 2 centile ranges (*n*)	8	6	
- Less than 3rd centile (*n*)	11	7	
Number of Patients Overweight (Weight ≥ 90%)	3	8	0.101
Mean Z-score of Patients with Failure to Thrive	−2.07	−2.08	
Mean Z-score of Patients without Failure to Thrive	−0.48	0.35	
Mean weight	13.9 kg	17.2 kg	0.002
Mean weight Z-score	−1.0	−0.18	0.001
Mean height Z-score	−0.67	−0.08	0.01

**Table 5:** Comparison of nutritional composition of the diets of “otherwise well” children with selective feeding as compared to children with autism spectrum disorder.

	“Otherwise Well” Children	Children with Autism Spectrum Disorder	*p*-Value
Inadequate calories (*n*, %)	29 (47.5%)	12 (20.3%)	0.001
Excessive calories (*n*, %)	1 (1.6%)	7 (11.8%)	0.061
Adequate calories but with insufficient micronutrients	10 (16.4%)	24 (40.7%)	0.003
No failure to thrive, but with insufficient calories and/or micronutrients	28 (45.9%)	25 (42.4%)	0.679
Overweight but with insufficient micronutrients	0 (0%)	2 (3.4%)	0.147
Milk as main source of calories	1 (1.6%)	9 (15.3%)	0.007

**Table 6:** Comparison of caregiver feeding styles of children with selective feeding based on CFSQ.

Caregiver Feeding Styles	“Otherwise Well” Children (38 Were Assessed)	Children with Autism Spectrum Disorder (49 Were Assessed)
Authoritarian (*n*, %)	22 (57.9%)	21 (42.9%)
Authoritative (*n*, %)	8 (21.1%)	10 (20.4%)
Indulgent (*n*, %)	4 (10.5%)	8 (16.3%)
Uninvolved (*n*, %)	4 (10.5%)	10 (20.4%)

**Table 7:** Comparison of caregiver responses of children with selective feeding.

Caregiver Response	“Otherwise Well” Children (35 Were Assessed)	Children with Autism Spectrum Disorder (47 Were Assessed)
Helplessness (*n*, %)	28 (80%)	34 (72%)
Frustration (*n*, %)	5 (14.3%)	1 (2%)
Anxiety (*n*, %)	3 (8.6%)	4 (8%)
Emerging (*n*, %)	10 (28.6%)	9 (15%)
Confidence (*n*, %)	0 (0%)	0 (0%)

Note: These descriptors are not mutually exclusive.

## Data Availability

Data are contained within the article.
